# Microencapsulation by spray-drying and freeze-drying of extract of phenolic compounds obtained from ciriguela peel

**DOI:** 10.1038/s41598-023-40390-4

**Published:** 2023-09-14

**Authors:** Marcony Edson da Silva Júnior, Maria Vitória Rolim Lemos Araújo, Ana Cristina Silveira Martins, Marcos dos Santos Lima, Flávio Luiz Honorato da Silva, Attilio Converti, Maria Inês Sucupira Maciel

**Affiliations:** 1 Food Science and Technology Graduate Program, Technology Center, Federal University of Paraíba, João Pessoa, Brazil; 2 Laboratory of Physical-Chemical Analysis of Food, Department of Consumer Sciences, Federal Rural University of Pernambuco, Recife, Brazil; 3 Department of Food Technology, Federal Institute of Sertão Pernambucano, Campus Petrolina, Rod. BR 407 Km 08, S/N, Jardim São Paulo, Petrolina, PE 56314-520 Brazil; 4 Department of Civil, Chemical and Environmental Engineering, Pole of Chemical Engineering, University of Genoa, Via Opera Pia 15, 16145 Genoa, Italy; 5 Food Science and Technology Graduate Program, Federal Rural University of Pernambuco, Recife, Brazil

**Keywords:** Chemistry, Materials science

## Abstract

Microcapsules of ciriguela peel extracts obtained by ultrasound-assisted extraction were prepared by spray drying, whose results were compared with those of freeze-drying as a control. The effects of spray-drying air temperature, feed flow rate and ratio of encapsulating agents (maltodextrin and arabic gum) were studied. Encapsulation efficiency, moisture content, total phenolic compounds (TPC), water activity, hygroscopicity, solubility, colorimetric parameters, phenolic profile by HPLC/DAD, simulated gastrointestinal digestion and morphology of spray-dried and freeze-dried microcapsules were evaluated, as well as their stability of TPC during 90 days storage at 7 and 25 °C. Spray-dried extract showed higher encapsulation efficiency (98.83%) and TPC (476.82 mg GAE g^−1^) than freeze-dried extract. The most abundant compounds in the liquid extract of ciriguela peel flour were rutin, epicatechin gallate, chlorogenic acid and quercetin. Rutin and myricetin were the major flavonoids in the spray-dried extract, while quercetin and kaempferol were in the freeze-dried one. The simulated gastrointestinal digestion test of microencapsulated extracts revealed the highest TPC contents after the gastric phase and the lowest one after the intestinal one. Rutin was the most abundant compound after the digestion of both spray-dried (68.74 µg g^−1^) and freeze-dried (93.98 µg g^−1^) extracts. Spray-dried microcapsules were of spherical shape, freeze-dried products of irregular structures. Spray-dried microcapsules had higher phenolic compounds contents after 90 days of storage at 7 °C compared to those stored at 25 °C, while the lyophilized ones showed no significant difference between the two storage temperatures. The ciriguela agro-industrial residue can be considered an interesting alternative source of phenolic compounds that could be used, in the form of bioactive compounds-rich powders, as an ingredient in pharmaceutical, cosmetic and food industries.

## Introduction

In Brazil, it stands out the consumption of ciriguela (*Spondias purpurea* L.), a species belonging to the genus *Spondias*, which comprises more than 600 species concentrated mainly in the tropical regions of Africa, Asia and Central America^1, 2^. From a phytochemical point of view, ciriguela is rich in secondary metabolites, mainly phenolic compounds^3^, the main of which are gallic acid, kaempferol, quercetin and isorhamnetin^4, 5^. Thanks to their important antioxidant effects, phenolic compounds are beneficial to human health; they can in fact act as potential agents to prevent and treat diseases related to the oxidative stress and even to control cancer^6^, in addition to having proven antibiotic, antiallergic, anti-inflammatory and photochemical protection effects^7^. In addition, they can be used as substitutes for synthetic antioxidants as well as to produce functional foods, drugs and cosmetics^8, 9^.

The main by-products of fruit processing are peels and seeds, whose extracts contain a considerable amount of valuable substances with potential application in food, cosmetic and pharmaceutical industries^10, 11^. Compared to other extraction methods, ultrasound assisted extraction stands out, because it is a simple, green and low cost process that uses acoustic energy to improve the release and diffusion of target compounds from various matrices, makes less use of solvents and requires lower temperatures and shorter extraction time^12–14^. There are numerous limitations in the application of these extracts due to the instability of bioactive compounds under conditions of high temperature and presence of oxygen, light and enzymes, as well as pH variations^15^. These bottlenecks can be overcome by microencapsulation techniques, which are capable of increasing the stability of these compounds by protecting them from environmental adverse effects thanks to the incorporation of a protective matrix^16, 17^.

Among them, spray-drying, or atomization, is the most popular microencapsulation method^18, 19^. This technique, which consists of transforming a fluid suspension into dry particles, is widely used to encapsulate heat-sensitive food ingredients, such as bioactive compounds, using an encapsulating agent that acts as a coating material^6, 17, 20^. Commonly used encapsulating agents are biopolymers such as maltodextrin with different dextrose equivalents and arabic gum. Variations in the quantity and type of coating agent result in different encapsulation efficiencies and in powders with different physical and chemical properties^21^.

However, no studies have examined the effect of ultrasonic extraction of phenolic compounds on ciriguela waste, as well the influence of temperature, feed flow rate, and ratio of encapsulating agents on spray microencapsulation. Given the above, the main objective of the present work was to study the influence of temperature, feed flow rate and ratio of encapsulating agents (maltodextrin and gum arabic) on the microencapsulation by spray-drying of ciriguela waste extracts prepared by ultrasound-assisted extraction. It was also compared the TPC stability of the spray-dried extract with that of a control powder prepared by freeze-drying over 90-day storage at different temperatures (7 and 25 °C), for its possible use as a functional ingredient in food, cosmetic and pharmaceutical applications.

## Materials and methods

### Materials

Ciriguela fruits were produced in the state of Paraíba (07° 09′ S 36º 49′ W). Ciriguela pulp by-products (peel and seed) were provided, after pulp extraction, by a frozen fruit pulp factory located in João Pessoa, PB, Brazil. After manual separation, 50 kg of ciriguela by-products yielded approximately 11 kg of ciriguela peel.

### Chemicals

All chemicals used in this study were obtained from Sigma-Aldrich (St. Louis, MI, USA) or Merck (Darmstadt, Germany).

### Preparation of ciriguela peel flour (CPF)

Ciriguela peel was subjected to drying at 60 °C for 24 h, according to Caldas et al.^13^, in an oven with air circulation and renewal (Model MA035/5, Marconi, Piracicaba, SP, Brazil) until reaching moisture below 10%. Then they were ground in a multipurpose mill (model TE 631/2, Tecnal, Piracicaba, SP, Brazil) and sieved through a 40-mesh (425 μm) screen. The resulting ciriguela peel flour (CPF) was stored in low-density 140-µm thick polyethylene bags, wrapped with laminated paper, and frozen to -20 °C for later analyses.

### Physicochemical and chemical characterization of ciriguela residue in natura and ciriguela peel flour

Soluble solids were determined with a digital refractometer (model r^2^i300, Reichert, Depew, NY, USA) and expressed in °Brix. The pH was analyzed using a pH-meter (HANNA, model HI 2210). The water activity (*a*_w_) was determined with a water activity meter (Decagon 4TE, Aqualab, Pullman, WA, USA) at 25 °C. The moisture content was determined on an infrared balance (model ID50, Marte Científica, São Paulo, SP, Brazil) at 105 ºC, and the results were expressed in %. Titrable acidity was determined titrimetrically, and the results were expressed in g of citric acid/100 g CPF^22^.

### Determination of total phenolic compounds in *natura* ciriguela residue and ciriguela peel flour

Ten g of CPF and 10 g ciriguela residue were separately extracted in 40 mL of solvent [80% (v/v) ethanol–water acidified with 0.1% (v/v) hydrochloric acid] by using a dynamic maceration, brought to room temperature (25 °C) and kept under stirring for 1 h. After extraction, suspended solids were removed by filtration through qualitative filter paper and the extracts obtained were stored at − 22 °C until further analysis.

The content of total phenolic compounds (TPC) in both *in natura* ciriguela residue and CPF was determined on a spectrophotometer (model UV-1650PC, Shimadzu, São Paulo/Brazil) at 725 nm using Folin-Ciocalteu reagent, according to the methodology described by Wettasinghe and Shahidi^23^. Briefly, 0.5 mL of each sample were incubated in a test tube with 8.0 mL of distilled water and 0.5 mL of Folin-Ciocalteu reagent. After 3 min of reaction, 1.0 mL of sodium carbonate solution was added, and the mixture was left to react for 60 min in the dark. The TPC content was calculated using a standard curve prepared from aqueous gallic acid solutions (0.1–1.0 mg/mL^−1^), and the results were expressed in mg of gallic acid equivalents (GAE) g^−1^ of CPF.

### Ultrasound-assisted extraction

Ten g of CPF with 40 mL of solvent [80% (v/v) ethanol–water acidified with 0.1% (v/v) hydrochloric acid] were placed in a 100 mL beaker and subjected to the action of a ultrasonic probe (model QR1000 Eco-sonic, Ultronique, Sao Paulo, SP, Brazil), using 1000 W power, 20 kHz frequency and 15 min exposure time^24^. The extracts obtained were filtered and kept in the absence of light at − 20 °C for later analyses.

### Microencapsulation of ciriguela peel flour extracts

#### Spray-drying

Microencapsulation by atomization was performed in a spray dryer (model MSD 1.0, Labmaq do Brasil, Ribeirão Preto, SP, Brazil). The carrier agents used were maltodextrin 10DE (Ingedion, Mogi-Guaçu, SP, Brazil) and gum arabic (Dinâmica Química Contemporânea, Indaiatuba, SP, Brazil). Extracts together with the encapsulating agent formulation were homogenized in Turrax (model TE-102, Tecnal—Piracicaba, São Paulo/Brazil) using a speed of 14,000 rpm for 5 min. The content of total solids in the suspension (wall material plus extract) was fixed at 30%, and atomization was performed using 1.2 mm diameter injector nozzle, 30 m^3^ h^−1^ air flow rate and 0.6 bar air pressure.

Runs were carried out according to a 2^3^-full experimental design composed of 8 factorial points (levels ± 1) and 3 central points (level 0), totaling 11 runs (Table 1).

**Table 1:** Matrix of the experimental design used for microencapsulation by spray-drying of ciriguela residue flour extract, with indication of the coded and real levels of the independent variables.

Run	Temperature (°C)	Ratio of encapsulating agents (%)	Feed flow rate (mL min^−1^)
1	− 1 (130)	− 1 (0% M/100% GA)	− 1 (0.40)
2	+ 1(170)	− 1 (0% M/100% GA)	− 1 (0.40)
3	− 1 (130)	+ 1 (100% M/0% GA)	− 1 (0.40)
4	+ 1 (170)	+ 1 (100% M/0% GA)	− 1 (0.40)
5	− 1 (130)	− 1 (0% M/100% GA)	+ 1 (0.80)
6	+ 1 (170)	− 1 (0% M / 100% GA)	+ 1 (0.80)
7	− 1 (130)	+ 1 (100% M / 0% GA)	+ 1 (0.80)
8	+ 1 (170)	+ 1 (100% M/0% GA)	+ 1 (0.80)
9	0 (150)	0 (50% M/50% GA)	0 (0.60)
10	0 (150)	0 (50% M/50% GA)	0 (0.60)
11	0 (150)	0 (50% M/50% GA)	0 (0.60)

M = maltodextrin; GA = gum arabic.

Temperature (*T*), ratio of encapsulating agents (*F*) and feed flow rate (*V*) were the independent variables, while moisture content, water activity, hygroscopicity, solubility, TPC content, encapsulation efficiency and chromatic parameters were the response variables. The data obtained were adjusted to Eq. (1):

1 $$Y = \beta_{0} + \beta_{1} T + \beta_{2} F + \beta_{3} V + \beta_{4} TF + \beta_{5} TV + \beta_{6} FV + \beta_{7} TFV$$

where *Y* is the response, *β*_0_ is the constant regression coefficient, *β*_1_, *β*_2_ and *β*_3_ are the linear coefficients, *T*, *F* and *V* are the independent variables, and *β*_4_, *β*_5_, *β*_6_ and *β*_7_ are the coefficients of the interaction effects *TF*, *TV*, *FV* and *TFV*, respectively.

#### Freeze-drying

Microencapsulation by freeze-drying, used as a control, was performed for 48 h in a lyophilizer (model Alpha 1–4 LD Plus, Martin Christ, Osterode am Harz, Germany) at − 80 °C and 0.28 mbar chamber pressure, using the optimum encapsulating agent’s formulation established for microencapsulation by spray-drying.

### Analysis of extracts microencapsulated by spray-drying and freeze-drying

#### Moisture content, water activity and total phenolic compounds content

These characteristics of the microencapsulated extracts were determined by the same methodologies used for *in natura* ciriguela residue and CPF.

#### Hygroscopicity

The hygroscopicity of microencapsulated extracts was determined according to the methodology described by Cai and Corke^25^ with some modifications. Briefly, one g samples of each microencapsulated extract were placed at 25 °C in an airtight container containing a saturated NaCl solution (75.29% relative humidity) and weighed after one week. Hygroscopicity was expressed in g of moisture absorbed per 100 g of sample dry mass (g/100 g).

#### Solubility

The solubility in water of microencapsulated extracts was determined according to the methodology reported by Cano-Chauca et al.^26^. To this purpose, 1.0 g samples of each powder were suspended in 100 mL of distilled water, stirred for 5 min in a magnetic stirrer (model 752, Fisatom, São Paulo, SP, Brazil) and centrifuged at 3000 rpm (1800 × *g*) for 5 min in a centrifuge (model CT-6000R, Cientec, Belo Horizonte, MG, Brazil). A 25-mL supernatant aliquot was placed in a pre-weighed sterilized Petri dish and kept at 105 °C for 5 h in oven. The plate was then weighed on an analytical balance, and the solubility determined by weight difference.

#### Chromatic parameters

The chromatic parameters of microencapsulated extracts were evaluated with a colorimeter (model CR 400, Konica Minolta, Sensing Inc., Osaka, Japan), using the color standards of the Commission Internationale de L'Eclairage (CIELab) system, namely, luminosity (L*) ranging from white (100) to black (0), intensity of the green (− a*) to red (+ a*) component of light, and intensity of the blue (− b*) to yellow (+ b*) component.

The variation in color (ΔE*) compared to the *in natura* extract was calculated by Eq. (2)^25^:

2 $$\Delta E* = \sqrt {\left( {L* - L_{0} } \right)^{2} + \, \left( {a* - a_{0} *} \right)^{2} + \, \left( {b* - b_{0} *} \right)^{2} }$$

where *L*_0_* and L*: are the luminosities of the samples of the free extract and the reconstituted microencapsulated extract, respectively; *a*_0_* and a*: are the red to green color intensities of the free extract and reconstituted microencapsulated extract samples, respectively; *b*_0_* and b*: are the intensities of the yellow to blue color of the samples of the free extract and the reconstituted microencapsulated extract, respectively.

#### Microencapsulation efficiency

The phenolics contents in microencapsules (TMPC) and on microcapsules surface (SMPC) were determined spectrophotometrically at 725 nm according to Saénz et al.^27^, after reaction with the Folin-Ciocalteu reagent and using a gallic acid standard curve^23^. The results were expressed in mg of gallic acid equivalents per g of microcapsules (mg GAE/g^−1^).

The microencapsulation efficiency (*ME*) was calculated by Eq. (3) according to Mahdavi et al.^28^:

3 $$ME \left( \% \right) = \frac{{{\text{TMPC }} - {\text{ SMPC}}}}{{{\text{TMPC}}}} \times 100$$

#### Apparent density

The apparent density of microcapsules (*ρ*_ap_) was determined according to the procedure described by Barbosa-Cánovas and Juliano^29^ and Caparino et al.^30^ with some modifications. Two g of sample were transferred to a 10-mL graduated test tube, where the powder was compacted by beating it 50 times on the bench. *ρ*_ap_, expressed in g/mL, was calculated according to Eq. (4):

4 $$\rho_{{{\text{ap}}}} = m/V$$

where *m* is the sample mass (g) and *V* the total volume occupied by the powder in the tube (mL).

#### Absolute density

The absolute density (*ρ*_abs_) was determined at 25 °C by means of a pycnometer provided with thermometer according to the methodology proposed by Caparino et al.^30^ and expressed in g mL^−1^.

#### Intragranular porosity

The intragranular porosity (*ε*) was calculated according to Caparino et al.^30^ using Eq. (5):

5 $$\varepsilon = \frac{{1 - \rho_{{{\text{ap}}}} }}{{\rho_{{{\text{abs}}}} }}$$

#### ***DPPH***^***.***^*** scavenging capacity***

The 1,1-diphenyl-2-picrylhydrazyl radical (DPPH^.^) scavenging capacity of microcapsules was assessed according to the method described by Brand-Williams et al.^31^ and modified by Sánchez-Moreno et al.^32^. The extract was diluted up to three different TPC concentrations by its addition to a 0.1 M DPPH: solution in methanol. The absorbance at 517 nm was monitored with a spectrophotometer (model UV-1650PC, Shimadzu, São Paulo, SP, Brazil) until the reaction reached a plateau. The results were expressed according to Ramadan et al.^33^. The inhibition percentage (IC_50_), i.e., the sample concentration needed to inhibit the DPPH• radical formation by 50%, was obtained according to Eq. (6):

6 $${\text{DPPH\% }} = { }\frac{{A_{{{\text{DPPH}}}} - A_{{\text{S}}} }}{{A_{{{\text{DPPH}}}} }} \times 100$$

where *A*_S_ is the absorbance of the sample suspension and *A*_DPPH_ is the absorbance of the DPPH solution.

The sample concentration providing IC_50_ was calculated by interpolation from the graph of radical-scavenging activity percentage against sample concentration.

#### Ferric reducing antioxidant power

The ferric reducing antioxidant power (FRAP) of microcapsules was determined by monitoring the absorbance at 593 nm, and the results were expressed as µmol of ferrous equivalent per g of powder (µmol Fe^2+^ g^−1^)^34^.

### Profile of phenolic compounds by HPLC–DAD

One g sample of microencapsulated extract was added to 10 mL of 6.0 M HCl in methanol and submitted for 30 min to extraction under ultrasonication at 25 °C and 40 kHz (model USC-1800, Unique, Indaiatuba, SP, Brazil). The extract was then centrifuged at 3000 × *g* for 20 min (model SL-701, Solab, Piracicaba, SP, Brazil). A 1.0-mL aliquot of the supernatant was filtered through a polytetrafluoroethylene syringe filter with 0.45-µm pore diameter and used to identify and quantify the phenolic compounds by High-Performance Liquid Chromatography (HPLC).

The methodology validated by Padilha et al.^35^, with adaptations by Dutra et al.^36^ on gradient and runtime, was used to quantify individual phenolic compounds using an Agilent 1260 Infinity LC System (Agilent Technologies, Santa Clara, CA, USA) coupled to a diode arrangement detector (DAD) (model G1315D). Separation of phenolic compounds was performed in a Zorbax Eclipse Plus RP-C18 column (100 × 4.6 mm, 3.5 µm) and a Zorbax C18 precolumn (12.6 × 4.6 mm, 5 μm). Data collection and analyses were carried out using the software OpenLAB CDS ChemStation Edition (Agilent Technologies). After dilution in solvent A (0.1 M phosphoric acid solution, pH 2.0), samples were filtered through a membrane with 0.45-μm pore diameter (Millex Millipore, Barueri, SP, Brazil) and injected (20 μL). The oven temperature was 35 °C, the eluent a mixture of solvents A and B (metanol acidified with 0.5% phosphoric acid), and the eluent flow rate 0.8 mL min^−1^. The gradient used in the separation was 0–5 min: 5% B, 5–14 min: 23% B, 14–30 min: 50% B, 30–33 min: 80% B. Detection of compounds was done at 220, 280, 320, 360 and 520 nm, and the identification and quantification by comparison with external standards. The results were expressed in µg g^−1^ dry weight.

### Simulated gastrointestinal digestion

The gastrointestinal digestion simulation was performed, according to Rodrigues-Roque et al.^37^ and Dutra et al.^4^, mimicking the physiological gastrointestinal conditions, i.e., considering two sequential phases in stomach (gastric) and small intestine including dialysis (intestinal). Microencapsulated extract aliquots (50 mL) were mixed with 5 mL of simulated salivary solution (2.38 g Na_2_HPO_4_, 0.19 g KH_2_PO_4_, 8 g NaCl, and 200 U/L of α-amylase) in amber vials. After homogenization of the mixture for 10 min in a water bath at 37 ± 1 °C and 95 × *g*, the samples were acidified to pH 2.0 with 1.0 mL of a porcine pepsin solution (13 mg pepsin in 5 mL of 0.1 M HCl) and later incubated at 37 °C under agitation at 95 × *g* for 1 h to simulate gastric digestion. The mixture was then immediately cooled in an ice bath, and a 1.0-mL aliquot was stored at − 18 °C, while the rest of the sample was submitted to intestinal digestion. Thirty cm long dialysis membrane segments were filled with 25 mL of a 0.5 M NaHCO_3_ solution, which was used to titrate the gastric digestate at pH 7.5. Each 20-mL sample of gastric digestate was placed in a polyethylene tube, and a dialysis membrane was completely immersed until pH 5.0 was reached. Then, 5.0 mL of pancreatine (0.12 g) and bile salts solution (40 mg glycodeoxycholate, 25 mg taurodeoxycholate, 40 mg taurocholate in 1.0 mL of saline) were added to each tube. The samples were incubated under agitation of 95 rpm at 37 °C for 2 h to complete the intestinal phase. Finally, the dialysis membrane was removed and rinsed with distilled water. The bioaccessible fraction of phenolic compounds transferred within the dialysis membrane was then analyzed by HPLC to determine the profile of residual phenolic compound after simulated gastrointestinal digestion.

### Average diameter and particle size distribution

The average diameter and particle size distribution were determined on a laser diffraction analyzer (model S3500, Microtrac, Largo, FL, USA) coupled to a common bench ultrasound device to increase sample dispersibility. A small amount of sample was dispersed in isopropyl alcohol as a carrying fluid and subjected to particle size distribution readings. The average diameter was determined based on the average diameter of a sphere of the same volume (Brouckere diameter, D[4.3]), which is generally used to characterize powder particles.

### Particle morphology

The morphology of microparticles was examined with a scanning electron microscope (SEM) (model Vega 3, Tescan, Brno, Czech Republic). The samples were fixed in metallic specimen holders (stubs) with a conventional electrically conductive double-sided adhesive tape, metallized with gold in a metallizer (model EM SCD500, Leica, Wetzlar, Germany) at a coating rate of 15 nm thickness for 80 s and a current of 40 mA, and examined with the above SEM operating at 10 kV. Image acquisition was performed using the XT microscope software.

### Storage stability of microcapsules

Microcapsules were stored according to Nunes et al.^38^ with some modifications. The samples (1.0 g) were placed in flexible laminated packages (Zip lock), kept at two different temperatures (7 and 25 ± 1 °C) and stored for 90 days. The TPC content was determined at 0, 15, 30, 45, 60, 75 and 90 days.

### Statistical analysis

Experimental data were analyzed and presented as means plus standard deviations of triplicate measurements. Analysis of variance (ANOVA), *F*-test for lack of fit, determination of regression coefficients and generation of response surfaces were done with the aid of Statistic 7.0 software (Statsoft, Tulsa, OK, USA) at 5% error probability.

## Results and discussion

### Characterization of the in natura ciriguela residue and ciriguela peel flour

Table 2 lists the results of the physicochemical characterization of *in natura* ciriguela residue and ciriguela peel flour (CPF).

**Table 2:** Physicochemical characterization of the *in natura* ciriguela residue and the ciriguela peel flour (CPF).

Parameter	*In natura* residue	CPF
Soluble solids (ºBrix)	0.90^b^ ± 0.01	4.63^a^ ± 0.15
pH	3.86^a^ ± 0.02	3.79^a^ ± 0.15
Water activity (*a*_w_)	0.983^a^ ± 0.001	0.178^b^ ± 0.004
Titratable acidity (g/100 g citric acid)	0.51^b^ ± 0.04	0.93^a^ ± 0.01
Moisture content (%)	69.57^a^ ± 2.26	5.83^b^ ± 0.15
Total phenolic compounds (mg GAE.g^−1^)	11.60^b^ ± 0.20	25.97^a^ ± 0.42

*Values are means ± standard deviation (n = 3). Different letters in the same line indicate significant differences among samples (*p* < 0.05). GAE = gallic acid equivalents.

The waste drying process resulted in a total solids content in CPF within the range found by other authors for flours of different fruit residues^39, 40^. The pH values of both *in natura* ciriguela residue and CPF are in the range of acid foods, which suggests inhibition of the growth of most pathogenic and deteriorating microorganisms. This value is in agreement with that reported by Albuquerque et al.^39^ (3.17) for ciriguela whole flour. The water activity (*a*_w_) of CPF (0.178 ± 0.004) was much lower than that of *in natura* residue, which indicates that it can be considered microbiologically safe^41^.

### Experimental design of ciriguela peel extract microencapsulation

Table 3 shows the results of the quality parameters (water activity, moisture content, hygroscopicity and solubility) of the CPFs prepared by spray-drying according to the experimental design shown in Table 1.

**Table 3:** Analyses of ciriguela peel flours prepared by spray-drying according to the experimental design shown in Table 1.

Run	Water activity (*a*_w_)	Moisture content (%)	Hygroscopicity (g/100 g)	Solubility (%)	*ME* (%)	TPC (mg GAE g^−1^ powder)
1	0.207^bcd^ ± 0.004	4.85^abc^ ± 0.17	13.99^b^ ± 0.28	86.43^b^ ± 0.91	99.14^ab^ ± 0.07	385.91^c^ ± 1.13
2	0.183^e^ ± 0.003	5.03^ab^ ± 0.04	12.16^de^ ± 0.22	87.84^ab^ ± 1.76	99.84^a^ ± 0.02	370.95^d^ ± 1.42
3	0.225^ab^ ± 0.001	4.23^cde^ ± 0.10	11.20^e^ ± 0.10	88.93^ab^ ± 0.21	93.47f. ± 0.44	190.45^j^ ± 0.56
4	0.200^cde^ ± 0.009	3.16f. ± 0.13	11.73^de^ ± 0.08	92.20^a^ ± 0.46	76.83^ g^ ± 0.76	218.48^ h^ ± 1.99
5	0.188^de^ ± 0.007	5.58^a^ ± 0.21	15.24^a^ ± 0.13	89.79^ab^ ± 2.00	98.40^bc^ ± 0.09	454.66^a^ ± 2.60
6	0.235^a^ ± 0.002	4.53^bcd^ ± 0.22	14.92^ab^ ± 0.24	88.77^ab^ ± 2.22	98.35^bc^ ± 0.09	432.69^b^ ± 1.42
7	0.155f. ± 0.002	3.65^ef^ ± 0.29	14.15^ab^ ± 0.39	89.39^ab^ ± 2.14	95.53^e^ ± 0.22	219.24^ h^ ± 1.42
8	0.225^ab^ ± 0.001	4.07^de^ ± 0.26	12.30^de^ ± 0.88	88.47^ab^ ± 0.99	98.23^c^ ± 0.12	201.82^i^ ± 0.56
9	0.218^abc^ ± 0.016	4.19^cde^ ± 0.26	12.57^d^ ± 0.13	88.02^ab^ ± 0.23	97.13^d^ ± 0.04	279.66^ g^ ± 0.98
10	0.210^bc^ ± 0.008	4.46^bcd^ ± 0.37	13.80^bc^ ± 0.52	89.49^ab^ ± 2.00	95.54^e^ ± 0.06	290.27f. ± 1.42
11	0.204^bcde^ ± 0.010	4.61^bcd^ ± 0.45	12.65^ cd^ ± 0.65	89.19^ab^ ± 1.93	97.68^ cd^ ± 0.02	296.89^e^ ± 2.15

*Values are means ± standard deviation (n = 3). Different letters in the same column indicate significant differences among powders (*p* < 0.05). *ME* = microencapsulation efficiency; TPC = total phenolic compounds content; GAE = gallic acid equivalents.

The results of analysis of variance (ANOVA) applied to linear models (Eq. 1) of each response variable, omitting the non-significant terms, are gathered in Table 4.

**Table 4:** Results of the analysis of variance (ANOVA) applied to linear models of response variables.

	Source					
	Water activity	Moisture content (%)	Hygroscopicity (g/100 g)	Solubility (%)	*ME* (%)	TPC (mg GAE.g^-1^ of powder)
Regression	0.014702*	12.57281*	49.46205*	57.8693	1219.093	263,356.6
Residual	0.002213	1.89926	7.46184	56.9365	30.531	4027.7
Lack of fit	0.000651*	0.17479^ ns^	0.90142^ ns^	1.1931^ ns^	21.391*	3261.4*
Pure Error	0.001562^ ns^	1.72447^ ns^	6.56042^ ns^	56.8998^ ns^	9.140^ ns^	506.3^ ns^
Total	0.016915	14.47207	56.92389	114.8058	1249.624	267,384.3
R-Squared	0.87	0.87	0.87	0.50	0.97	0.98

ns: Not significant (*p* > 0.05). *Significant at (*p* < 0.05)*; ME* = microencapsulation efficiency; TPC = total phenolic compounds contents; GAE = gallic acid equivalents.

As is known, *a*_w_, moisture content and hygroscopicity are important characteristics for the stability and storage of any powder, while solubility is associated with its eventual reconstitution. All *a*_w_ values of spray-drying microencapsulated extracts (0.155–0.235) (Table 3) were lower than the threshold limit (0.3) below which powders can be considered stable and resistant to the attack of both spoilage microorganisms and enzymes responsible for lipid oxidation^42, 43^. *a*_w_ values close to those obtained in this study were reported for different bioactive compounds-rich microencapsulated extracts^19, 44–47^.

This response was significantly influenced by inlet air temperature (*T*), interaction between temperature and feed flow rate (*TV*) and interaction between the ratio of encapsulating agents and feed flow rate (*FV*), according to Eq. (7):

7 $$a_{{\text{w}}} = 0.{2}0{5} + 0.00{8}T + 0.0{2}0TV - 0.00{9}FV$$

**Figure 1:**
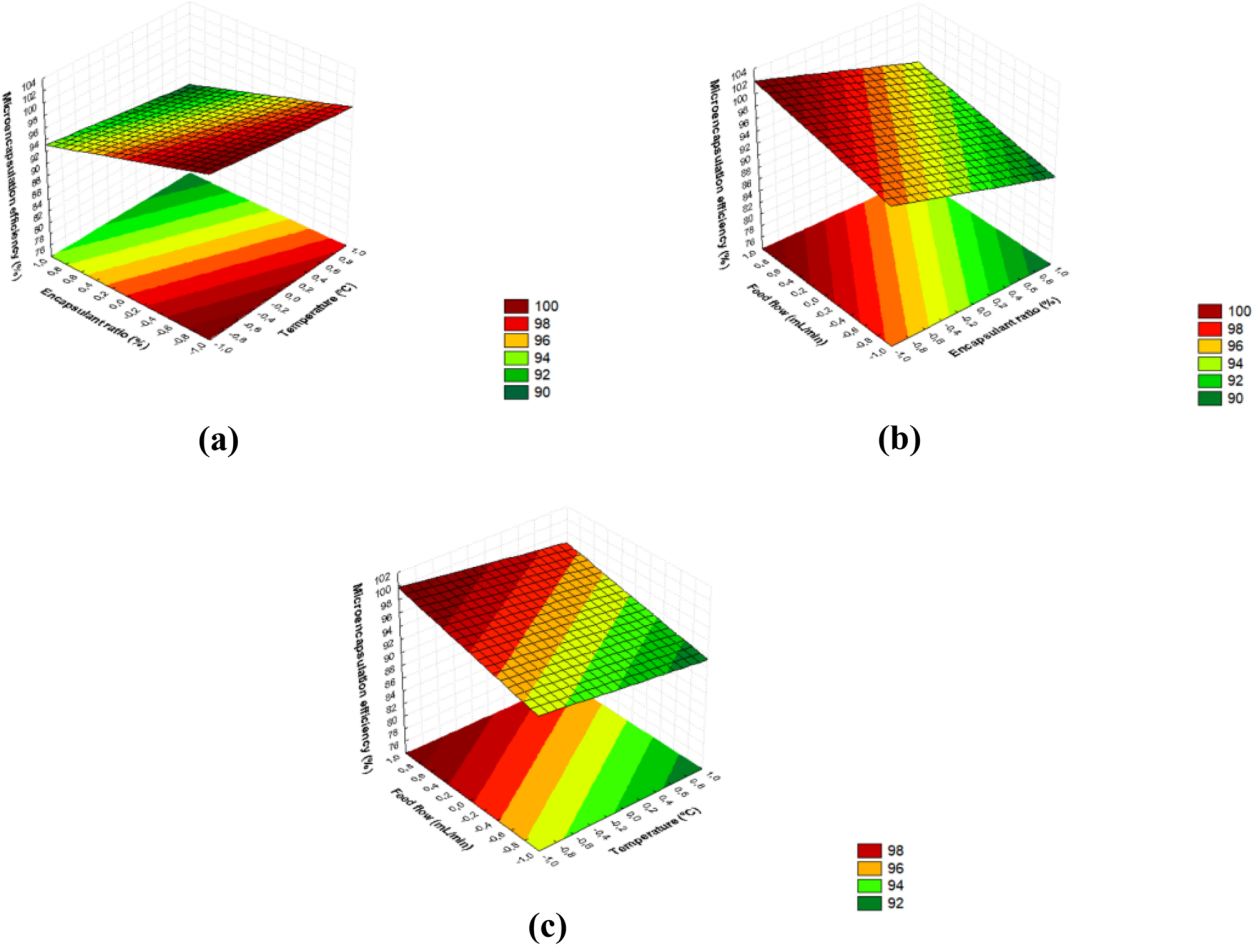
Response surfaces of the efficiency of ciriguela residue extracts encapsulated by spray-drying as a function of (**a**) temperature and ratio of encapsulating agents; (**b**) feed flow rate and ratio of encapsulating agents; and (**c**) feed flow rate and temperature.

The higher temperature, the lower the *a*_w_ value of powder extracts, which indicates its positive effect on this response, similar to that observed by Tülek et al.^48^ for atomized lemongrass extract. When using a high feed flow rate of (0.80 mL min^−1^) and combined with 100% gum arabic as an encapsulating agent, the extracts showed lower *a*_W,_ probably due not only to the influence of the *F*x*V* and *T*x*V* binary (*p* < 0.05) (Figs. 1S and 2S of the Supplementary Material), but also the ability of gum arabic to incorporate water molecules thanks to the presence of proteins and hydrophilic groups in its composition^19^. Similarly, Bednarska and Janiszewska-Turak^49^ and Janiszewska-Turak et al.^43^ observed that the greater the addition of gum arabic, the lower the aw values of chokeberry and carrot powder juices, respectively.

Moisture contents in the 1–6% range are sought by industry to ensure stability of powder products during storage^42^. All moisture contents obtained in this study, varying from 3.16 to 5.58% (Table 3), fell within this range and were close to those reported in several studies for different atomized fruit extracts or by-products, such as cagaita (*Eugenia dysentrica* DC)^50^, grape (*Vitis labrusca var*. Bordo) skin^45^ and grape pomace^51^, acerola residue^47^, pineapple peel^52^, maca leaf^16^, cranberry juice^19^, cocoa pod husk^46^, and mulberry (*Morus alba* L.) leaf^6^.

Moisture content was significantly influenced by the linear effects of *T* and *F* as well as the ternary interaction between *T*, *F* and *V* (*p* < 0.05) (Tables 3 and 4), according to Eq. (8):

8 $${\text{Moisture}}\;{\text{content}} = {4}.{39} - 0.{18}T - 0.{61}F + 0.{33}TFV$$

ANOVA showed a coefficient of determination (R^2^) of 0.87 (Table 4), indicating that fitting of experimental data to this model was more than satisfactory. In particular, the decrease in microcapsule moisture content resulting from an increase in air inlet temperature (Figs. 3S of the Supplementary Material). The temperature had a negative effect on the moisture of the powders, higher inlet air temperature (170 °C) produced extracts in powders with lower moisture contents, probably due to different drying rates between the droplets of the feed solution and the air of drying due to their different physical properties^52^. The same negative effect of air temperature on moisture content has been reported for different phenolic compounds-rich atomized extracts^50–53^. According to Kaderides et al.^54^, the higher the temperature difference between drying air and particles, the higher the heat transfer rate to the particles, thus causing a driving force for moisture removal. The powders produced with gum arabic as an encapsulating agent exhibited the highest moisture contents and (Fig. 3S of the Supplementary Material), confirming the trend observed by Pudziuvelyte et al.^18^ for *Elsholtzia ciliata* essential oil microcapsules. According to Tran and Nguyen^55^, gum arabic has higher capacity of absorbing water from surrounding environments than maltodextrin.

The hygroscopicity of powders, varying from 11.20 to 15.24 g of water absorbed/100 g of sample (Table 3), was low, which promises to facilitate their conservation and preserve their color and content of bioactive compounds. This response variable was significantly influenced by *T*, *F*, *V* and ternary *T*x*F*x*V* interaction (*p* < 0.05) (Tables 3 and 4), according to Eq. (9):

9 $${\text{Hygroscopicity}} = {13}.{15} - 0.{43}T - 0.{86}F + 0.{94}V - 0.{48}TFV$$

Hygroscopicity values close to those of this study were reported for different spray-dried extracts^44, 45, 47, 50^. More hygroscopic powders were produced when higher feed rates were used. This response was influenced mainly and positively by *V* and negatively by *F* (Figs. 4S, 5S and 6S of the Supplementary Material). Similar results were obtained for chokeberry juice powder^49^.

Water solubility of atomized extracts, ranging from 86.43 to 92.20% (Table 3), fell within the solubility range reported for other atomized fruit extracts or by-products^18, 44, 47, 52^. It was significantly influenced by *F* and by the *F*x*V* and *T*x*V* binary interactions (*p* < 0.05), according to Eq. (10):

10 $${\text{Solubility}} = {88}.{96} + 0.{77}F - 0.{82}TV - 0.{94}FV$$

This model, however, was not predictive due to a too low R^2^ value (0.5) (Table 4), so the effect of each variable on powders solubility were illustrated in the form of Pareto Graph (Fig. 7S of the Supplementary Material). The maltodextrin/gum arabic ratio in the coating agents formulation had a positive effect on water solubility, in that, powders produced with maltodextrin were more soluble, although the solubility of all them was high (Fig. 8S of the Supplementary Material). The high solubility of powders produced in this work can be related to the high solubility of the selected encapsulating agents, as well as the product granulometry, in that, the lower the particles size, the greater the surface area available for hydration^45, 56^. As shown in Fig. 9S* (Supplementary Material),* the most soluble microencapsulated extracts were those produced using the lowest feed flow rate and maltodextrin as a wall material.

The microencapsulation efficiency (*ME*) of atomized extracts ranged from 76.83 to 99.84% (Table 3), indicating that the selected formulation of encapsulating agents was very efficient in protecting phenolic compounds. This response was significantly influenced by all independent variables and their combinations (*p* < 0.05) according to Eq. (11):

11 $$ME = 95.46 - 1.66T - 3.95F + 2.65V - 1.82TF + 2.32TV + 3.21FV + 2.51TFV$$

The model had statistical and predictive significance with R^2^ of 0.97. When the value of pure error is low as in this case, i.e., the reproducibility is very good, the lack of fit, even though significant (Table 4), is a false result due to the fact that the *F*-calculated value is high because of the very low denominator value. This model was validated through ANOVA before building the response surface charts illustrated in Fig. 1a–c.

The spray-dried extracts produced with gum arabic showed the highest *ME* values, in agreement with the results obtained in microcapsules of *E. ciliata* essential oil^55^. It has been reported that *ME* of microencapsulated extracts would be related to the combination of encapsulating agents used, which would be responsible for different filmogenic properties, keeping the active principle protected^45^.

The TPC content of spray-dried extracts, which ranged from 190.45 to 454.66 mg GAE g^−1^ (Table 3), was significantly influenced by *F*, *V*, *TF*, *TV*, and *FV* (Table 4) according to Eq. (12):

12 $${\text{TPC}}\,{\text{content}} = {3}0{3}.{72}{-}{1}0{1}.{77}F + {17}.{82}V + {5}.{94}TxF{-}{6}.{55}TxV - {14}.{79}FxV$$

**Figure 2:**
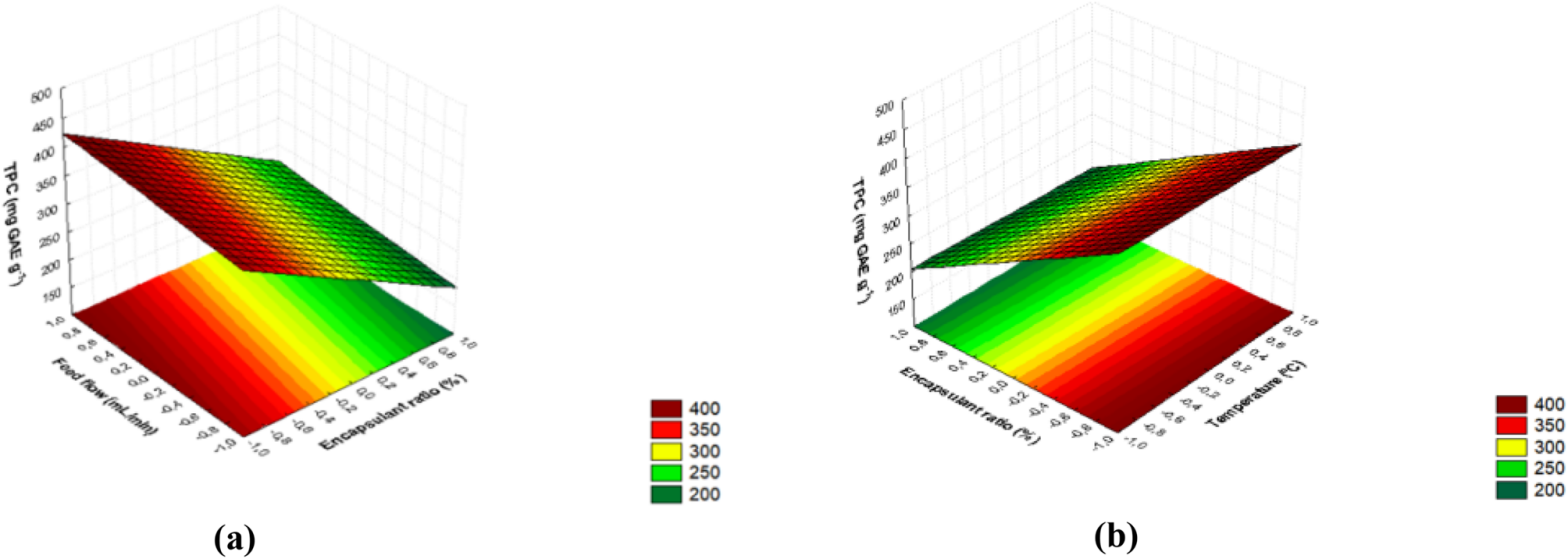
Response surfaces of the total phenolic compounds (TPC) content of ciriguela residue extracts encapsulated by spray-drying as a function of (**a**) ratio of encapsulating agents and feed flow rate and (**b**) ratio of encapsulating agents and temperature.

The high R^2^ value (0.98) compensates for the significant lack of fit for the same reasons as for *ME* (Table 4), indicating that the model has statistical and predictive significance. The influence of independent variables on this response is illustrated by the response surfaces in Fig. 2a and b.

Since the ratio of coating agents in the encapsulating formulation was the variable that most influenced this response, followed by the *FV* interaction, the highest TPC contents were obtained using gum arabic (Fig. 2a and b). A similar result was reported for the spray-drying of grape (*V. labrusca* var. Bordo) skin extract^45^. This special gum arabic capacity of encapsulating TPC has been related to its structure, being a highly branched sugar heteropolymer with small protein content, which allows protection of these compounds mainly during the critical early phase^45, 47^. As a result, all the runs performed using the mixture of encapsulating agents (9, 10, and 11) also led to TPC contents higher than those obtained only with maltodextrin, similar to what was observed by Rezende et al.^47^ for atomized acerola residue extract.

### Selection of the best conditions for ciriguela residue extract spray-drying and freeze-drying

According ANOVA applied to results from runs performed according to the above experimental design, the optimal conditions for microencapsulation by spray-drying of CPF extracts were a temperature of 150 °C, a feed flow rate of 0.80 L h^−1^ and 100% gum arabic as a coating agent. Microencapsulation by freeze-drying, used as a control, was performed with the same encapsulating agent. Table 5 lists the results of physicochemical parameters and antioxidant activity of ciriguela residue extracts microencapsulated by both drying methods.

**Table 5:** Physicochemical parameters and antioxidant activity of ciriguela residue extracts microencapsulated by spray-drying and freeze-drying.

Parameter	Spray-drying*	Freeze-drying*
TPC content (mg GAE g^−1^)	486.82^b^ ± 3.04	532.96^a^ ± 0.58
Microencapsulation efficiency (%)	98.83^a^ ± 0.07	88.76^b^ ± 0.42
DPPH % (IC_50_)	68.21^a^ ± 0.59	48.84^b^ ± 0.59
FRAP (µmol Fe^2+^ g^−1^)	4.826,48^a^ ± 49.65	4.802,01^a^ ± 23.41
Moisture content (%)	4.51^a^ ± 0.12	4.35^a^ ± 0.20
Solubility (%)	89.02^a^ ± 1.37	86.37^a^ ± 2.36
Hygroscopicity (g 100 g)	14.83^a^ ± 0.07	14.84^a^ ± 0.30
L*	89.73^a^ ± 0.39	88.42^b^ ± 0.38
a*	− 0.07^a^ ± 0.03	− 0,05^a^ ± 0.03
b*	13.07^b^ ± 0.33	17,11^a^ ± 0.36
Δ*E* *	55.04^a^ ± 2.04	53.17^a^ ± 1.67
Apparent density (g mL^−1^)	0.25^b^ ± 0.01	0.31^a^ ± 0.01
Absolute density (g mL^−1^)	1.06^a^ ± 0.01	1.07^a^ ± 0.01
Intragranular porosity (%)	70.44^a^ ± 0.86	64.37^b^ ± 0.95

*Values are means ± standard deviation (n = 3). Different letters in the same column indicate significant differences among powders (*p* < 0.05). TPC = total phenolic compounds content; GAE = gallic acid equivalents; DPPH = antioxidant activity by the DPPH method; IC_50_ = concentration needed to inhibit the DPPH• radical formation by 50%; FRAP = antioxidant activity by the ferric reducing antioxidant power method; L* = luminosity; a* = intensity of green/red component of light; b* = intensity of yellow/blue component of light; ΔE* = difference in color.

*ME*, TPC content, antioxidant activity by the DPPH method, apparent density and intragranular porosity showed statistically significant differences (*p* < 0.05) between spray-dried and freeze-dried extracts, while antioxidant activity by the FRAP method did not (*p* > 0.05) (Table 5). These results suggest the importance of using different methods for the safe and conclusive determination of antioxidant activity, as each method has its own specificities and acts better in a specific field^47^. Higher values of *ME*, TPC content and antioxidant activity by DPPH were obtained for the spray-dried extract (*p* < 0.05). On the other hand, Ramírez et al.^57^, when studying the stability of freeze-dried and spray-dried fruits, observed greater retention of polyphenols in the former encapsulates.

Luminosity (L*) values were close to 90.00 (Table 5), indicating that spray-dried and freeze-dried extracts had color close to white, similarly to what was observed for spray-dried cagaita extract^50^. According to Comunian et al.^58^, these results are due to the dilution effect of the white encapsulating agents. Although microencapsulation by spray-dring, due to high inlet air temperature, can lead to darkening reactions responsible for color loss^59^, the intensity of green/red component of light (a*) and difference in color (ΔE*) showed no significant difference between the two extracts, unlike the intensity of yellow/blue component of light (b*) (*p* < 0.05). Taking the colorimetric parameters as a whole (L*, a*, b*), it can be concluded that the CPF extract microcapsules can be considered of greenish-yellow color like those obtained from mulberry (*M. alba* L.) leaf^6^.

Physical parameters showed no significant differences between spray-dried and freeze-dried extracts (*p* > 0.05), except for apparent density and intragranular porosity that were 24% higher (0.31 ± 0.01 g mL^−1^) and 8.6% lower (64.37 ± 0.95%), respectively, for the freeze-dried extract (Table 5). It is noteworthy that these characteristics play an important role in controlling the degree of rehydration and reconstitution of powders.

### Phenolics profiles in liquid and microencapsulated extracts and during simulated gastrointestinal digestion

The profile of phenolic compounds was investigated by HPLC–DAD in both the liquid extract and the microcapsules prepared by spray-drying and freeze-drying using 100% gum arabic as a coating agent (Table 6). Phenolic acids, flavanols and flavonols were identified as the four major groups, while flavanones, stilbenes and anthocyanins were detected in lower amounts. Although the contents of identified phenolics varied statistically between the two powders (*p* < 0.05), as expected, their qualitative profile (Table 6) was relatively similar to that observed for frozen ciriguela pulp^4^ and ciriguela peel^60, 61^. This shows that after microencapsulation of ciriguela residue extract, the microcapsules still had phenolic compounds with biological activities of interest such as antioxidant, antimicrobial and anti-inflammatory attributed to the various phenolic compounds found^4, 62^.

**Table 6:** Phenolics profile in liquid (control), spray-dried and freeze-dried ciriguela residue extracts.

Phenolic compound (µg g^−1^)	Retention time (min)	Liquid extract (control)	Spray-dried extract	Freeze-dried extract
Phenolic acids				
*trans*-Caftaric acid	13.4	14.09ª ± 0.26	ND	ND
Chlorogenic acid	14.6	228.31ª ± 5.67	40.09^b^ ± 1.53	17.72^c^ ± 0.17
Caffeic acid	17.1	18.01ª ± 3.03	ND	ND
*p*-Coumaric acid	23.3	15.45ª ± 0.62	ND	ND
Flavanols				
Procyanidin B1	12.6	5.96^b^ ± 0.12	ND	16.74ª ± 0.22
Procyanidin B2	16.9	15.47ª ± 0.37	ND	ND
Catechin	14.6	60.95^b^ ± 0.93	16.59^c^ ± 0.07	93.94ª ± 0.67
Epicatechin	19.3	105.71ª ± 2.02	22.40^b^ ± 0.06	ND
Epigallocatechin gallate	20.2	28.96^b^ ± 0.48	53.62ª ± 0.99	ND
Epicatechin gallate	25.3	228.63ª ± 12.78	71.23^b^ ± 0.41	41.85^c^ ± 1.02
Procyanidin A2	26.4	80.40ª ± 4.70	83.70ª ± 4.28	35.08^b^ ± 0.55
Flavonols				
Myricetin	23.9	65.85ª ± 0.24	21.60^b^ ± 0.52	17.15^b^ ± 0.40
Rutin	25.3	342.59ª ± 45.08	82.69^b^ ± 0.70	54.66^c^ ± 0.35
Quercetin	25.7	181.02ª ± 6.28	11.75^c^ ± 0.08	26.77^b^ ± 0.90
Kaempferol	27.0	5.32^c^ ± 0.02	12.84^b^ ± 0.26	70.46ª ± 1.33
Isorhamnetin	27.9	ND	54.78ª ± 0.54	ND
Flavanones				
Hesperidin	27.1	92.48ª ± 5.09	80.34^b^ ± 2.07	ND
Stilbenes				
*trans*-Resveratrol	29.2	26.38ª ± 0.86	ND	ND
Anthocyanins				
Petunidin 3-glucoside	24.7	7.19ª ± 1.45	ND	ND

*The results are expressed as mean ± standard deviation (n = 3). Values expressed in µg g^−1^ dry matter. Means followed by the same letters in the same line do not differ by Tukey’s test at 5% error probability. ND—not detected.

Rutin (342.59 ± 45.08 µg g^−1^), epicatechin gallate (228.63 ± 12.78 µg g^−1^), chlorogenic acid (228.31 ± 5.67 µg g^−1^), and quercetin (181.02 ± 6.28 µg g^−1^) were the most abundant phenolic compounds in the CPF liquid extract. Chlorogenic acid was the only phenolic acid found in spray-dried (40.09 ± 1.53 µg g^−1^) and freeze-dried (17.72 ± 0.17 µg g^−1^) extracts. Catechin content was significantly higher (*p* < 0.05) in the freeze-dried extract (93.94 ± 0.67 µg g^−1^) compared to both liquid and spray-dried extracts (Table 6). Isorhamnetin was present only in the spray-dried sample, while epicatechin, epigallocatechin gallate, and hesperidin were only detected in the liquid and spray-dried extracts. Rutin was the major flavonol in both liquid (342.59 ± 45.08 µg g^−1^) and spray-dried (82.69 ± 0.70 µg g^−1^) extracts, while kaempferol was in the freeze-dried one (70.46 ± 1.33 µg g^−1^). Santana Neto et al.^63^ observed a similar phenolic acids profile to that of this study in extract ciriguela residue, and among the flavonoids identified, rutin had the highest level.

*trans*-Caftaric acid, caffeic acid, *p*-coumaric acid, *trans*-resveratrol, and petunidin 3-glucoside were found only in the liquid extract. In this regard, it has been reported that when fruits are subjected to processing, including maceration, crushing, microencapsulation, or freezing, oxidation and/or degradation of antioxidant compounds may occur^4, 64–66^.

The contents of phenolic compounds recovered after each phase (oral, gastric, and intestinal) of simulated gastrointestinal digestion of microencapsulated extracts are listed in Table 7. It is noteworthy that the higher this residual content, the greater the resistance of microcapsules to gastric conditions. The highest values were found after the gastric phase (*p* < 0.05), while the minor ones after the latter, i.e., the intestinal one. A similar result was reported for flour from persimmon (*Diospyros kaki*) fruit co-product during in vitro gastrointestinal digestion^67^.

**Table 7:** Phenolic compounds contents (µg g^−1^, mean values ± standard deviation, n = 3) obtained during simulated gastrointestinal digestion of spray-dried and freeze-dried ciriguela residue extracts.

Compound (µg g^−1^)	Sample	Phase		
		Oral	Gastric	Intestinal
Chlorogenic acid	Spray-dried	ND	36.06^B^ ± 2.39	ND
	Freeze-dried	ND	45.29^A^ ± 0.32	ND
Procyanidin B1	Spray-dried	ND	11.80^B^ ± 0.91	ND
	Freeze-dried	ND	14.67^A^ ± 0.46	ND
Catechin	Spray-dried	ND	32.99^B^ ± 0.95	ND
	Freeze-dried	ND	35.53^A^ ± 0.02	ND
Epicatechin	Spray-dried	ND	11.22^B^ ± 0.22	ND
	Freeze-dried	ND	95.45^A^ ± 0.57	ND
Epigallocatechin gallate	Spray-dried	ND	29.49^A^ ± 0.26	ND
	Freeze-dried	ND	ND	ND
Epicatechin gallate	Spray-dried	33.10^b^ ± 0.57	50.69^Ba^ ± 0.98	ND
	Freeze-dried	ND	59.98^Aa^ ± 8.41	17.10^Ab^ ± 0.27
Procyanidin A2	Spray-dried	ND	ND	37.90 ± 1.66
	Freeze-dried	ND	ND	ND
Rutin	Spray-dried	23.61^Bb^ ± 0.20	68.74^Ba^ ± 1.97	ND
	Freeze-dried	28.74^Ab^ ± 0.64	93.98^Aa^ ± 2.00	21.24^c^ ± 0,16
Quercetin	Spray-dried	ND	66.53^A^ ± 3.17	ND
	Freeze-dried	ND	10.64^B^ ± 0.27	ND

*Different superscript lowercase letters on the same line indicate a statistically significant difference (*p* < 0.05) for the same compound obtained by the same microencapsulation method at different stages of gastrointestinal digestion. Different superscript capital letters in the same column indicate a statistically significant difference (*p* < 0.05) based on t-test, for the same compound at the same stage of gastrointestinal digestion obtained by different microencapsulation methods. µg g^−1^: micrograms of phenolic compound per gram of dry matter; n: number of samples; ND: No detection.

While in the post-gastric phase most phenolic compounds were identified, in the post-intestinal phase there was a significant reduction in the number of compounds. This tendency may be related to digestion conditions, mainly due to changes in gastric pH (pH 2.0) and intestine (pH 7.5)^68^. The acidic conditions of the gastric phase probably contributed to the release of the compounds.

During gastric digestion, several phenolic acids, flavanols, and flavonols were detected, highlighting the significant phenolic content available for absorption. However, more research is needed to investigate the dynamics of the absorption of phenolic compounds in the stomach. The transition from gastric to intestinal digestion reduced the bioaccessibility of most compounds, but did not increase the bioaccessibility of procyanidin A2 from the atomized extract.

Regarding the microcapsule production method, freeze-drying ensured greater phenolic compound content than spray-drying, except for epigallocatechin gallate and quercetin. Most phenolic compounds were completely degraded after the gastric phase, except for epicatechin gallate and rutin in the freeze-dried extract, while procyanidin A2 was detected only after the intestinal phase in the spray-dried one. Finally, the highest concentrations of epicatechin gallate and rutin (*p* < 0.05) were detected after the gastric phase, similar to what was observed for persimmon fruit co-product flour^67^.

A total of three compounds were detected after the intestinal phase of digestion and six were not detected. This finding is consistent with several studies that found a considerable decrease in phenolic compounds after gastrointestinal digestion^67, 69, 70^. The loss of these compounds may have been caused by conditions in the intestinal environment that were unfavorable to its stability, such as alkaline pH, pancreatin, and bile secretions^71^. After the intestinal phase, the presence or absence of phenolic compounds revealed the variable chemical behavior of phenolic compounds. For example, quercetin, which is extracted in relatively large amounts during gastric digestion, is not detected after intestinal digestion. A similar result was found in the study by Nignpense et al.^70^. This is consistent with evidence that quercetin has low solubility in alkaline media such as water and intestinal juice^72^.

The chemical characteristics of phenolic compounds, such as solubility, hydrophobicity, molecular weight, or even isomer configuration, are influencing their stability in the course of digestion^73^. The decrease of most phenolic compounds during in vitro digestion, regardless of the FRC extract microencapsulation method, may be related to the release of proteins and fibers by enzymatic degradation. Since both proteins and carbohydrates are able to interact with phenolic compounds and block their detection through spectrophotometric analysis^68, 74^. Changes in the simulated digestive environment’s pH can convert anthocyanins and transform some phenolics into different structural formulas with various chemical properties^75^.

### Average diameter and particle size distribution

The particle size of powder extracts has been associated with important characteristics such as susceptibility to deterioration, fluidity, appearance and dispersibility^76^. Particle size distribution graphs (Fig. 10S of the Supplementary Material) allowed to calculate for spray-dried and freeze-dried microcapsules average diameters (16.75 and 25.19 µm, respectively) within the values reported for spray-drying in general (10–100 µm)^77^, and for other microencapsulated natural products or by-products^38, 47, 78, 79^. The largest size of freeze-dried particles compared to the spray-dried ones may have been due to the low process temperature as well as the lack of strength to break frozen drops or to change surfaces during drying)^80^.

### Particle morphology

**Figure 3:**
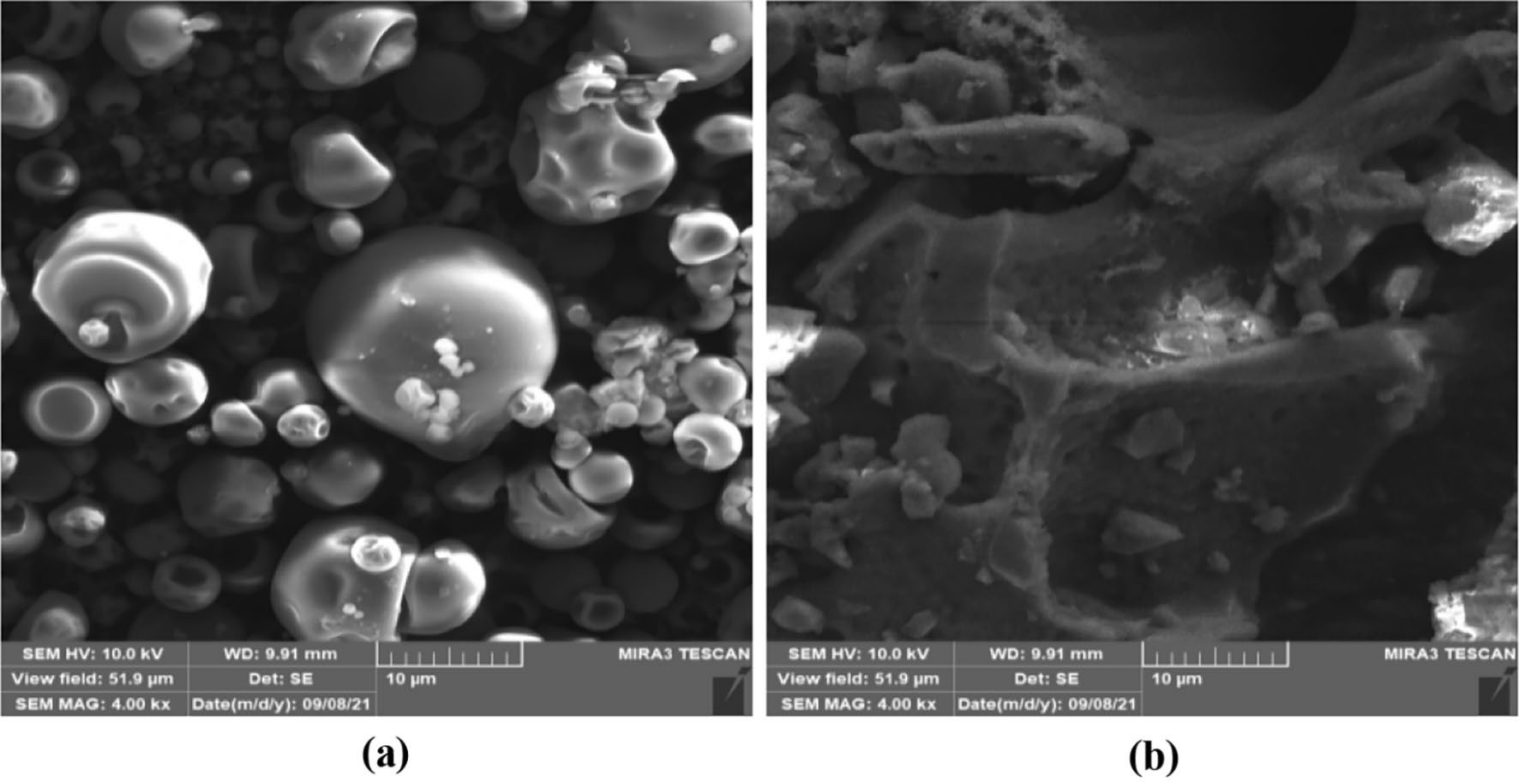
Scanning electron micrographs of ciriguela peel extract microparticles prepared by (**a**) spray-drying and (**b**) freeze-drying.

Figure 3 shows the scanning electron micrographs of CPF extracts microencapsulated by spray-drying (a) and freeze-drying (b). Microcapsules prepared by freeze-drying had a more deformed and irregular shape, with extensive wrinkles and a more toothed surface than the ones prepared by spray-drying, which had a spherical shape, few deformations, and a smooth surface, confirming the observations of other authors^38, 50, 81^.

According to Ballesteros et al.^82^, the quite different conditions of spray-drying and freeze-drying result in different morphology, shape, and size of microparticles. Moreover, superficial imperfections, such as wrinkles or cracks, occur when there is slow film formation during droplet microencapsulation^83^. Also, the type of encapsulating agent can influence the morphology of microcapsules. In fact, Bernstein and Noreña^84^ obtained similar particles with the toothed surface using gum arabic, likely due to sudden moisture loss during microencapsulation^81^. Even the deformations of microcapsules, i.e., irregular spherical shape, shrinks, and wrinkles on their surface, may have been due to the use of arabic gum as an encapsulating agent^50^.

### Storage stability of microcapsules

**Figure 4:**
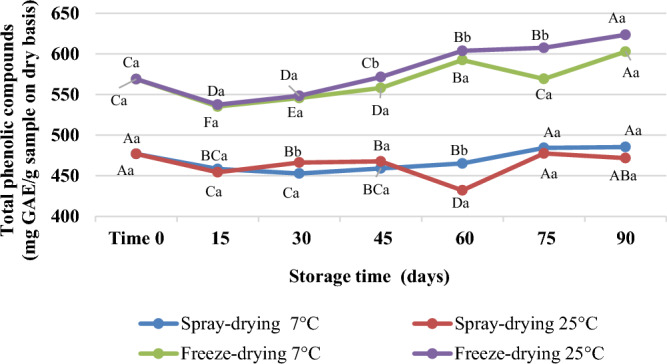
Stability analysis of spray-dried and freeze-dried microcapsules of ciriguela residue extract stored at 7 and 25 °C for 90 days in terms of ability to retain phenolic compounds. Different capital letters for microcapsules stored at the same temperature indicate statistically significant differences over time for the same powder. Different lowercase letters indicate statistically significant differences between powders obtained by the same encapsulation method and stored at different temperatures.

Microcapsules placed in flexible laminated packages were stored for 90 days at 7 ± 1 °C, aiming at their use as an additive for low temperature stored products, and 25 ± 1 °C, representing the room temperature. Figure 4 shows the stability of atomized and lyophilized microcapsules under these conditions in terms of ability to retain their TPC content over time.

The freeze-dried microcapsules of CPF extract did not show any statistically significant difference in their TPC content (602.77–623.41 mg GAE g^−1^) (*p* > 0.05) after 90 days compared to the beginning (568.86 ± 0.98 mg GAE g^1^), regardless of the storage temperature. The spray-dried powder extract started with 476.82 ± 2.04 mg GAE g^−1^, and at the end of the stability at 7 °C it presented 485.34 ± 1.50 mg GAE g^−1^ and at 25 °C, 471.70 ± 2.47 mg GAE g^−1^, so there was no significant difference between the start and end time. This small increase in TPC after stability is due to recoveries and the formation of polyphenols as a consequence of the hydrolysis of conjugated polyphenols^38, 85^.

## Conclusions

Spray-drying and freeze-drying proved to be suitable processes to prepare ciriguela peel extract microcapsules to be used as a source of phenolic compounds for foods, such as drinks, salty cookies, cookies, bakery products among others, as well as pharmaceuticals and cosmetics. The optimized conditions of microencapsulation by atomization were shown to be a temperature of 150 °C, a feed flow rate of 0.80 L h^−1^ and the use of 100% gum arabic as an encapsulating agent. The spray-dried extract showed higher TPC (486.82 mg GAE g^−1^). The phenolic compounds found in greater concentrations in the liquid extract of ciriguela residue were rutin, epicatechin gallate, chlorogenic acid, and quercetin, while rutin and myricetin were the most abundant in the atomized extract, and quercetin and kaempferol in the freeze-dried one. After simulated gastrointestinal digestion of microencapsulated extracts, rutin was the phenolic compound most abundant in microcapsules. Spray-dried and freeze-dried microcapsules, regardless of storage temperature (7 or 25 °C), maintained their high content of phenolic compounds almost unvaried for 90 days of storage.

### Supplementary Information

Supplementary Information.

## Data Availability

The data used to support the findings of this study are available from the corresponding author upon request.
