# Supplementary material for: Microencapsulation by spray-drying and freeze-drying of extract of phenolic compounds obtained from ciriguela peel
Source: Sci Rep. 2023 Sep 14;13:15222. doi: 10.1038/s41598-023-40390-4 (PMC10502068; doi:10.1038/s41598-023-40390-4)
Supplement: Supplementary file 1 — Supplementary Information. [file 41598_2023_40390_MOESM1_ESM.docx]

**Microencapsulation by spray-drying and freeze-drying of extract of phenolic compounds obtained from ciriguela peel**

**Marcony Edson da Silva Júnior^a^, Maria Vitória Rolim Lemos Araújo^b^, Ana Cristina Silveira Martins^a^, Marcos dos Santos Lima^c^, Flávio Luiz Horonato da Silva^a^, Attilio Converti^d^, Maria Inês Sucupira Maciel^a^***

a Food Science and Technology Graduate Program, Technology Center, Federal University of Paraíba, João Pessoa, Brazil.

b Laboratory of Physical-Chemical Analysis of Food, Department of Consumer Sciences, Federal Rural University of Pernambuco, Recife, Brazil.

c Federal Institute of Sertão Pernambucano, Department of Food Technology, Campus Petrolina, Rod. BR 407 Km 08, S/N, Jardim São Paulo, 56314-520, Petrolina, PE, Brazil.

d Department of Civil, Chemical and Environmental Engineering, University of Genoa, Pole of Chemical Engineering, Via Opera Pia 15, I-16145 Genoa, Italy.

*Corresponding author (Maria Inês S. Maciel). E-mail address: m.inesdcd@gmail.com

Fone: + 55 81 994381078/ 33206536 / Fax: +55 81 33206540

**Supplementary Material**


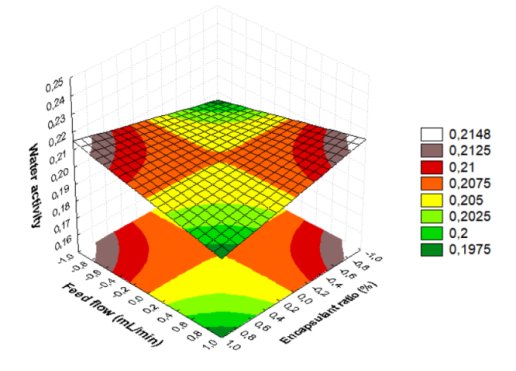


**Figure 1S.** Response surface of water activity of ciriguela residue extract encapsulated by spray-drying as a function of feed flow rate and ratio of encapsulating agents.


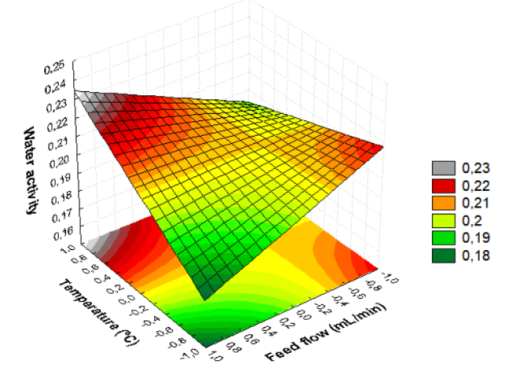


**Figure 2S.** Response surface of water activity of ciriguela residue extracts encapsulated by spray-drying as a function of temperature and feed flow rate.


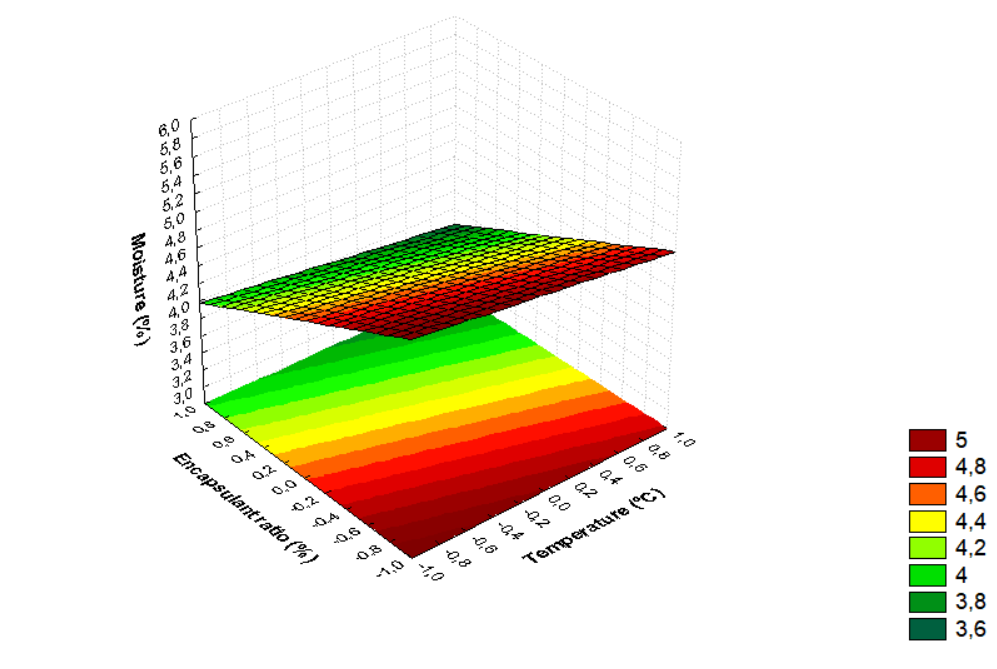


**Figure 3S.** Response surface of moisture content of ciriguela residue extract encapsulated by spray-drying as a function of temperature and ratio of encapsulating agents.


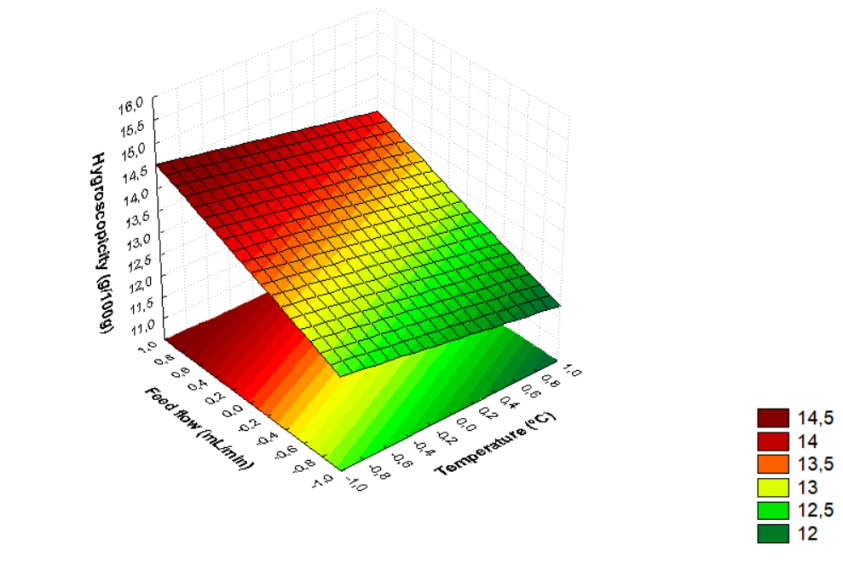


**Figure 4S.** Response surface of hygroscopicity of ciriguela residue extract encapsulated by spray-drying as a function of feed flow rate and temperature.


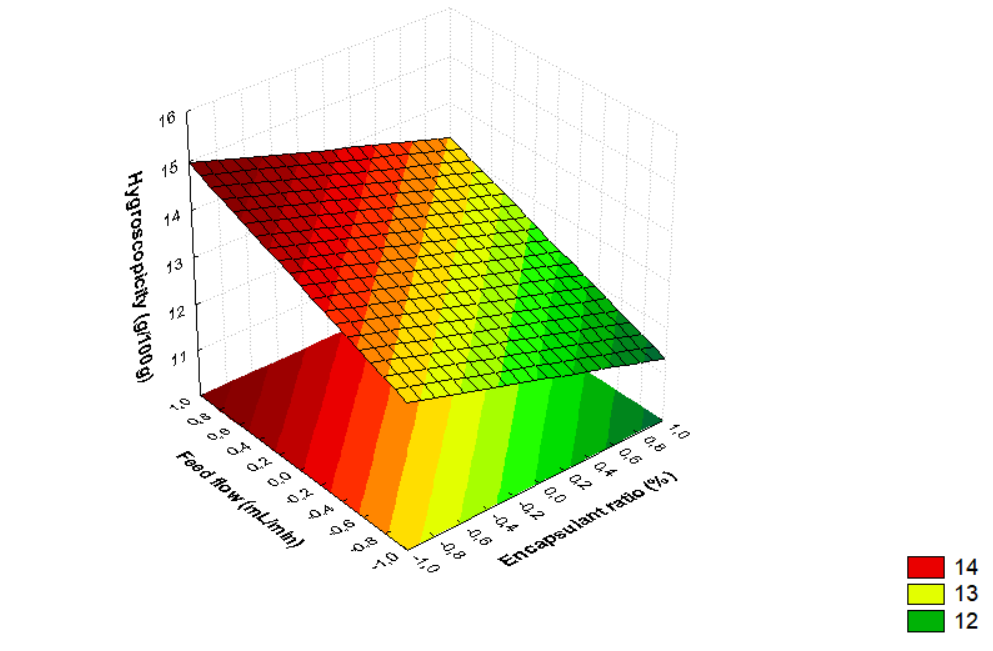


**Figure 5S.** Response surface of hygroscopicity of ciriguela residue extract encapsulated by spray-drying as a function of feed flow rate and ratio of encapsulating agents.


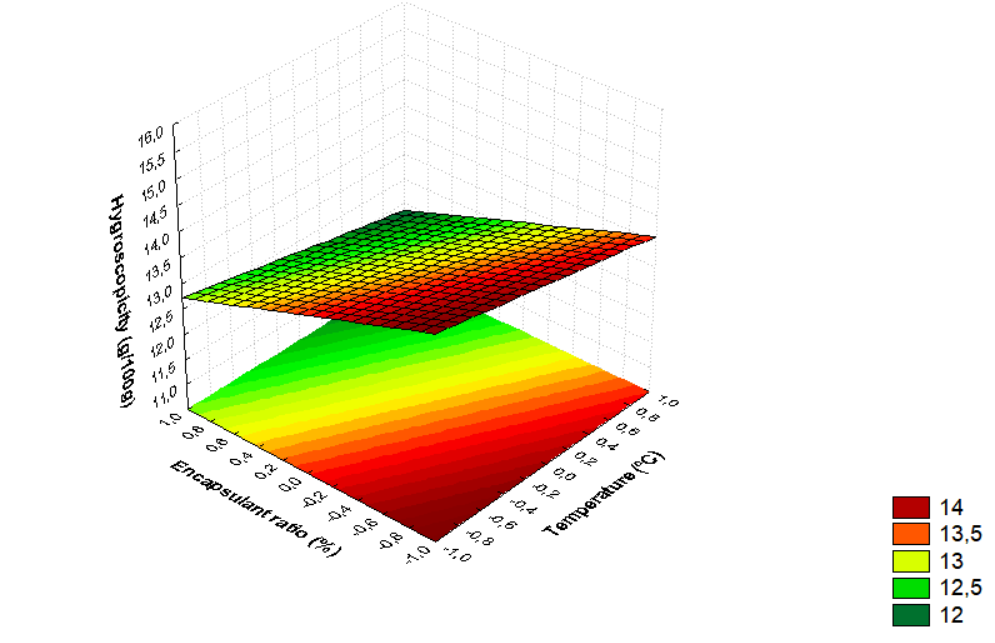


**Figure 6S.** Response surface of hygroscopicity of ciriguela residue extract encapsulated by spray-drying as a function of temperature and ratio of encapsulating agents.

**Figure 7S.** Pareto Chart of the solubility of ciriguela residue extract encapsulated by spray-drying.


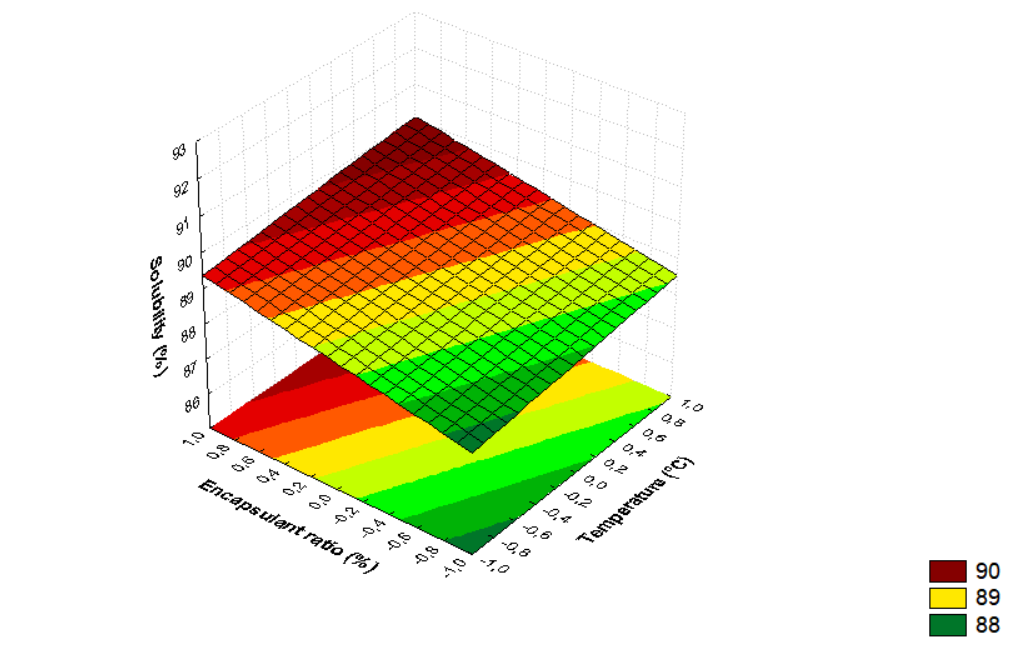


**Figure 8S.** Response surface of solubility of ciriguela residue extract encapsulated by spray-drying as a function of ratio of encapsulating agents and temperature.


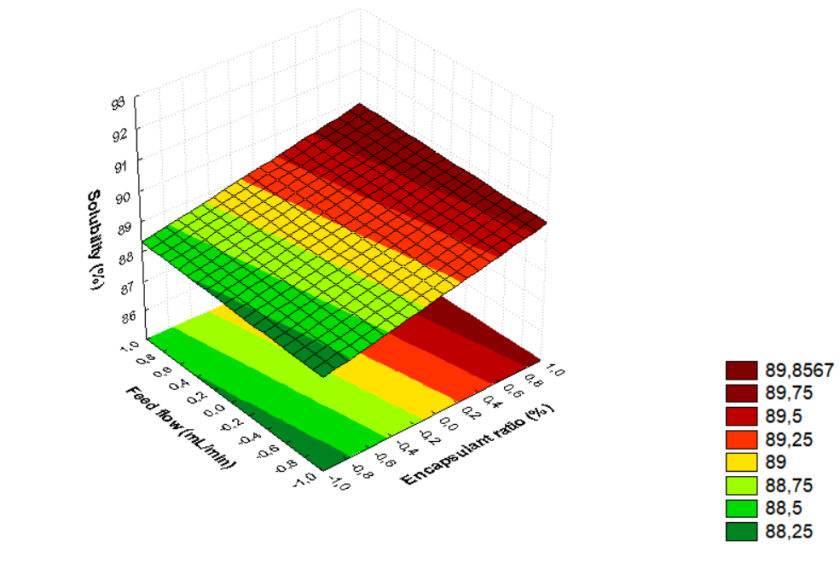


**Figure 9S.** Response surface of solubility of ciriguela residue extract encapsulated by spray-drying as a function of feed flow rate and ratio of encapsulating agents.

*
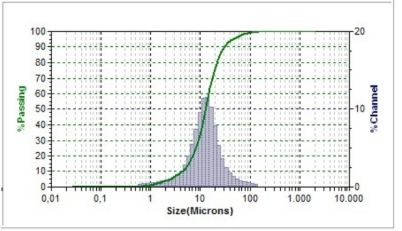

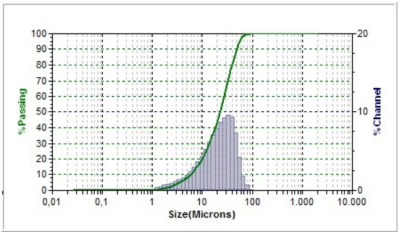
*

**(a) (b)**

**Figure 10S.** Particle size distribution of ciriguela residue extracts encapsulated by (a) spray-drying and (b) freeze-drying.
